# Quality of Diabetes Care: The Challenges of an Increasing Epidemic in Mexico. Results from Two National Health Surveys (2006 and 2012)

**DOI:** 10.1371/journal.pone.0133958

**Published:** 2015-07-31

**Authors:** Sergio Flores-Hernández, Pedro J. Saturno-Hernández, Hortensia Reyes-Morales, Tonatiuh Barrientos-Gutiérrez, Salvador Villalpando, Mauricio Hernández-Ávila

**Affiliations:** 1 Centro de Investigación en Evaluación y Encuestas, Instituto Nacional de Salud Pública, Cuernavaca, Morelos, México; 2 Dirección del Centro de Investigación en Evaluación y Encuestas, Instituto Nacional de Salud Pública, Cuernavaca, Morelos, México; 3 Dirección de Investigación, Hospital Infantil de México “Federico Gómez”, México, Distrito Federal; México; 4 Centro de Investigación en Salud Poblacional, Instituto Nacional de Salud Pública, Cuernavaca, Morelos, México; 5 Centro de Investigación en Salud y Nutrición, Instituto Nacional de Salud Pública, Cuernavaca, Morelos, México; 6 Dirección General, Instituto Nacional de Salud Pública, Cuernavaca, Morelos, México; McMaster University, CANADA

## Abstract

**Background:**

The quality of diabetes care remains suboptimal according to numerous studies assessing the achievement of quality indicators for diabetes care in various healthcare settings. We report about global and specific quality indicators for diabetes care and their association to glycemic control at the population level in two national health surveys in Mexico.

**Methods:**

We conducted a cross-sectional analysis of the 2006 and 2012 National Health Surveys in Mexico. We examined quality of care for 2,965 and 4,483 adults (≥ 20 years) with diagnosed type 2 diabetes using fourteen simple and two composite indicators derived from self-reported information. In a subsample for both surveys, glycated hemoglobin (HbA1c) was measured at the time of the interview. We obtained survey weight-adjusted estimators using multiple regression models (logistic and linear) with combined data files, including survey year as covariate to assess change.

**Results:**

Global quality of care in 2012 was 40.8%, with a relative improvement of 11.7% between 2006 and 2012. Detections of cardiovascular disease risk factors (dyslipidemia and hypertension) were the indicators with the highest improvement, while non-pharmaceutical treatment and diabetic foot exams showed minor changes. We found a significant association between the quality of the process of diabetes care and glycemic control (OR 2.53, 95% CI 1.63-3.94). Age more than 65 years old, the type of health subsystem, gender (males), and high socio-economic status were also significantly associated to glycemic control.

**Conclusions:**

Quality diabetes care and glycemic control improved and are significantly associated. However, according to international standards, the current situation remains suboptimal. A more holistic approach is needed, with an emphasis on improving quality in outpatient care.

## Introduction

Diabetes is an increasingly prevalent global health problem. Worldwide an estimated 8.3% of adults -387 million people- suffer diabetes, the majority of them living in developing countries, and 90% of adults have Type 2 diabetes [1,2]. In the Organization for Economic Cooperation and Development (OECD) countries, the estimated prevalence of diabetes in adults aged 20 to 79 is 6.9% [3]. Mexico ranks among the top 10 countries in the world for Type 2 diabetes [1], with a prevalence rate of 9.1% in 2012, representing 6.4 million patients diagnosed [4]. It remains as the 9^th^ leading cause of death and the highest in burden of disease measured by adjusted life years, with a 90% increase between 1990 and 2010 [5]. The health care costs Type 2 diabetes and its complications are also high. In 2010, the costs for diabetes health care in Mexico were estimated at 3.43 billion dollars [6], while reducing life expectancy by an average of 5–10 years. At the same time, Type 2 diabetes is a major cause of ambulatory care consultations. In Mexico City, causes over 13 million visits a year or 41.5% of the visits for chronic non-communicable diseases, particularly Type 2 diabetes [7].

There is evidence that quality diabetes care prevents or delays complications [8]. National and International guidelines have been developed to support diabetes care management. However, National Surveys in different countries document important deficits in the quality of diabetes care [9–12], of which remain a challenge for the health care system [13,14].

Quality of care can be measured in terms of process of care including regular glycated hemoglobin (HbA1c) testing, as well as intermediate outcome measure such as achievement of glycemic control. In addition, international standards [15,16] recommend the control of risk factors (lipid levels, high blood pressure and avoiding tobacco) and detection of potential complications (retinopathy, nephropathy, etc.) early in the disease process to achieve the best health outcomes [17,18].

In view of the magnitude of the problem, the National Health Surveys have investigated various aspects of the care received by patients with diagnosed diabetes to improve understanding of this problem at the population level. The objective of the present analysis is to use the data provided by these surveys to assess the quality of the outpatient care provided to Mexican adults with Type 2 diabetes and associated glycemic control.

## Methods

### The Mexican context

Most of the Mexican population is affiliated to the public health care system, which is organized in several different and autonomous sub-systems: social security serving the population who work in the formal vector and their families (IMSS), social security for public servants (ISSSTE), social security for workers in other governmental branch, such as the Mexican Oil Industry (PEMEX), and public health insurance offered by the Ministry of Health to persons without social security (*Seguro Popular*); a low proportion of population receive health care services from private providers, and may be affiliated to private health insurance. In spite of all these schemes, close to 20% of the population remains uncovered by any insurance. This population receives care from the network of public institutions owned and managed by the Ministry of Health and its counterparts in the states, which also serve the people affiliated to the *Seguro Popular*.

Results from the 2000 National Health Survey reported poor indications of health with respect to Type 2 diabetes, including poor compliance for important care issues such as cardiovascular disease risk factor detection and adequate non-pharmacologic and pharmacologic treatment [19]. In response, multiple uncoordinated initiatives have been implemented the last decade in the different public subsystems to address better care for chronic conditions focused particularly in diabetes [20, 21]. However partial evaluations carried out in different segments of the population consistently reveal that diabetes care remains suboptimal [22, 23]. The 2006 and 2012 National Health Surveys collected more information than previous surveys regarding diabetes care, including a blood test to measure HbA1c in a subsample of surveyed persons with diagnosed diabetes. This provided the opportunity to asses both quality of care and the level of glycemic control at population level.

### Data sources and selection criteria

A secondary analysis was carried out on data obtained from the 2006 and 2012 National Health and Nutrition Surveys (ENSANUT). Surveys details are described elsewhere and the data are publicly available [24, 25]. Briefly, both surveys were cross-sectional and used stratified multistage probability cluster sampling techniques to collect representative population data at national, regional and state level. The sampling procedure included a randomized selection of households, stratified by clusters, from the National Household Sampling Frame designed by the National Institute of Statistics, Geography and Informatics. These surveys collected health and nutrition information, as well as socio-demographic data from 48,304 and 50,528 households and from 45,446 and 46,303 adults ≥20 years, with response rates of 80% and 87% respectively. This design makes both rounds of survey data comparable. Additionally, the formulation of the questions regarding diabetes care for a structured interview questionnaire was the same for most variables.

For this study, we analyze data from adults (≥20 years) who self-reported a previous diagnosis of Type 2 diabetes (2,965 and 4,483 in ENSANUT 2006 and 2012 respectively). The gateway question was “*Has a doctor told you that you have diabetes or high blood sugar*?". Women diagnosed with gestational diabetes were excluded. HbA1c levels were measured at the time of the 2006 and 2012 surveys in a subsample of respondents with diabetes. Blood samples (2006: n = 1,093; 2012: n = 750) were analyzed in a central laboratory.

All participants signed an informed consent prior to completing the surveys. The National Health Surveys and the consent forms were approved by the Ethics, Research and Biosecurity Committees of the National Institute of Public Health, Mexico.

### Variables

Variables of the patients surveyed included age and gender, time since diagnosis, presence of diabetes-related complications (leg or foot ulcers, amputation, decreased vision, retinal damage, loss of sight, dialysis, vascular complications, diabetic coma, foot pain or burning), cardiovascular disease risk factors (tobacco use, obesity, dyslipidemia, and hypertension), cardiovascular disease (heart attack, angina, heart failure or other heart disease), and other co-morbidities (depression, chronic obstructive pulmonary disease, asthma, musculoskeletal disease, neurological disease, and cancer).

To assess diabetes ambulatory care quality, we analyzed four components: (1) diabetes monitoring and control; (2) detection and surveillance of cardiovascular disease risk factors; (3) prevention of vascular complications; and (4) treatment (non-pharmacological and pharmacological). Each component included one or more indicators according to international [15–17] and national [26] standards of care. Relevant questions for the survey instruments were derived from previously selected quality of diabetes care indicators, particularly in relation to ambulatory of process of care making this analysis possible. The complete list include 10 process of care indicators applicable to all adults with Type 2 diabetes (during the past year: visiting a care provider four or more times, HbA1c test at least two times, checking for high blood pressure, dyslipidemia, overweight/obesity, and protein in urine, eyes and foot exams, and treatment (non-pharmacologic and pharmacologic), and four additional indicators for those who also self-reported hypertension and/or dyslipidemia (controlling blood pressure, lipid profile, and treatment non-pharmacologic and pharmacologic).

The quality score for each indicator is the frequency of compliance with the quality of care criteria expressed as a percentage over eligible respondents. For instance, if 4,483 respondents were eligible for a given quality care criterion, and indicated care was given in 2,900 of them, the quality score would be (2,900/4,483) x 100 = 64.7%.

To summarize overall quality of care we use two composite indicators [27–28]: “percentage of overall quality” and “percentage of comprehensive quality” (compliance with all the applicable indicators). The "*percentage of overall quality*" is obtained by dividing the total number of compliances (sum of the number of compliances of each specific indicator) by the total number of cases for which each indicator was assessed, multiplied by 100 [(Σ compliances of each indicator/Σ of occasions wherein each indicator is assessed) X 100]. This composite indicator was estimated for: (i) all 14 indicators (global); (ii) the 10 general indicators (general, applying to all respondents with Type 2 diabetes); and (iii) the four indicators that apply only to those with an additional cardiovascular disease risk factor (overall additional).

Glycemic control was defined by individual HbA1c targets based on age and presence of complications (existing retinopathy, nephropathy, or cardiovascular disease): for those younger than 45 years without complications ((≤ 6.5%) [≤ 47.5 mmol/mol]), and with complications ((≤ 7.0%) [≤ 53 mmol/mol]); for those aged 45 to 64 years without complications ((≤ 7.0) [≤ 53 mmol/mol]), and with complications ((≤ 8.0%) [≤ 63.9 mmol/mol]); and for those aged 65 or more years without complications ((≤ 7.5%) [≤ 58.5 mmol/mol]), and with complications ((≤ 8.0%) [≤ 63.9 mmol/mol]) [15–18, 27] by using American Diabetes Association recommendations [15–18, 29].

Socioeconomic-status (SES) was measured using a previously validated index [30], which was constructed estimating per capita consumption and expenditure level using the data and approach from the 2006 and 2012 National Household Income and Expenditure Surveys.

### Data Access

Available Data in: http://ensanut.insp.mx/

Access to the database (Bases de datos y documentación) held by National Public Health Institute, Center for Evaluation and Survey, ENSANUT, requires the completion of a free user registration (Formulario de Registro). One time registration will provide a username and password via e-mail in order to access all relevant data.

Link to Registration (Formulario de registro): http://ensanut.insp.mx/forma_registro.php#.VZLNK_l_Oko

### Data Analysis

First, we estimated changes in the prevalence of diagnosed Type 2 diabetes in adults and their characteristics, comparing indicators of compliance between 2006 and 2012. Changes in quality of care were assessed in absolute and relative terms. Absolute change was calculated as the difference in percentages of compliance between 2012 and 2006. Relative change (or *relative improvement*) was calculated as follows: (absolute improvement/possible room for improvement)x100 = [absolute improvement/(100-percentage of compliance in 2006)]x100 [31]. For the outcome indicator we assessed changes between 2006 and 2012 in the mean HbA1c, the proportion of adults diagnosed with diabetes who met individual HbA1cs, and the percentages of three categories of HbA1c level (<7%, 7–9% and >9%).

To assess adjusted changes between the two surveys and the influence of quality process criteria on glycemic control, we used a combined 2006 and 2012 data file creating new weights using jackknife replication method, with R = 80 replicate weights for each survey year, given that each ENSANUT samples are drawn independently and with different weights. In this combined file, ENSANUT 2006 is year 1 and ENSANUT 2012 year 2. The analysis followed the methodology described by Lee S, et al. [32], taking into account the complex sample design of both surveys, to obtain unbiased estimates. Statistical significance of changes between 2006 and 2012 were considered at the 5% significance level calculating p-value using the student-*t* distribution and by computing 95% confidence intervals (CI), (Wald test and/or “*lincom* post-estimation command”).

To assess the relationship between quality of care and glycemic control, we used regression models (multiple linear and logistic), where glycemic control was the dependent variable, and the quality of care indicators (global composite or individually) as the independent variables. We included in the models the following adjusting covariates: survey year (1 or 2), gender (male = reference), age (<45 years = reference), time since diagnosis of diabetes (≤10 years = reference), presence of diabetes-related complications, presence of cardiovascular disease risk factors, type of health subsystem (affiliation to a public medical insurance *(Social Security* = reference) and SES by quintile (quintile I, the lowest = reference). To test if the composite global quality of process indicator was associated to glycemic control, we restricted the data set to the eligible group, using the subpopulation estimation for survey data (the “svy” command with “subpop” option) for observations with HbA1c measurements. In the multiple linear regression model HbA1c is expressed as percentage; we analyzed the adjusted association with the percentage of global quality care. In the logistic regression model HbA1c is expressed as achievement of individualized target based on age and presence of complications (retinopathy, nephropathy, or cardiovascular disease) and we calculated adjusted Odds ratios (OR) with 95% CI to identify significant associations.

All statistical analyses were performed using Stata software version 13.0 (Stata, Stata Corp, College Station, TX).

## Results

### Changes in prevalence and characteristics of patients with Type 2 diabetes

The National Surveys showed a significant increase in the prevalence of people who knew they suffered from Type 2 diabetes (7.0% in 2006 to 9.1% in 2012); the estimated change between years was 2.1% (95% CI 1.5–2.7%), (data not shown in table).

Table 1 shows the results of estimated changes between 2006 and 2012 in relevant patient characteristics (age and gender, presence of complications due to diabetes, cardiovascular disease risk factors, co-morbidities, and time since diagnosis) as well as the level of glycemic control. The percentages of adults with diagnosed Type 2 diabetes who self-reported complications (62.9% in 2012), presence of cardiovascular disease risk factors (80.7% of adults with diabetes in 2012), and mean time since diagnosis, all increased significantly. Glycemic control levels showed significant improvements, with fewer surveyed patients with HbA1c > 9% [>75 mmol/mol] in 2006 (72.8%) than in 2012 (50.3%), an estimated change of -22.5 points. There was also a higher percentage of adults with diagnosed diabetes who met individualized HbA1c target in 2012 (29.7% compared to 4.0% in 2006.

**Table 1:** Characteristics and glycemic control of adults with diabetes: Changes between ENSANUT (2006 and 2012).

	Adults n = 45,241	ENSANUT 2006	Adults n = 46,277	ENSANUT 2012	Estimated Change 2012–2006
	n	Weighted (95% CI)	n	Weighted (95% CI)	Weighted % (95% CI)
**Adults with diagnosed diabetes**	n = 2,965		n = 4,483		
Diabetes + complications^†^	1,608	54.4% (54.1–54.6)	2,809	62.9% (62.3–63.5)	8.5* (7.9–9.1)
Diabetes + CVD Risk Factors^‡^	2,402	80.7% (80.5–81.0)	3,809	86.9% (86.5–87.2)	6.1* (5.7–6.5)
Diabetes + comorbidities^§^	512	17.7% (17.5–17.9)	720	17.4% (16.9–17.9)	-0.3 (-0.8–0.3)
Age, mean (years)	2,965	56.2 (56.1–56.3)	4,483	56.9 (56.6–57.0)	0.7 (0.5–0.9)
% Females	1,793	57.5% (57.2–57.8)	2,760	55.5% (54.9–56.2)	-2.0 (-2.6–1.3)
Time since diagnosis of diabetes Mean (years)	2,965	8.3 (8.2–8.3)	4,450	9.2 (9.1–9.3)	0.9* (0.8–1.0)
**% HbA1c**	n = 1,093		n = 750		
Mean		11.5 (11.2–11.8)		9.3 (9.0–9.6)	-2.2* (-2.6 to -1.7)
**HbA1c levels**					
< 7% (53 mmol/mol)	55	3.5% (2.3–4.8)	164	25.6% (20.0–31.2)	22.1* (16.3–27.8)
7–9% (53–75 mmol/mol)	275	23.7% (20.1–27.3)	206	24.1% (19.6–28.7	0.4 (-5.4–6.3)
>9% (>75 mmol/mol)	763	72.8% (69.0–76.6)	381	50.3% (44.6–55.9)	-22.5* (-29.3 to -15.7)
**Glycemic control** ^¶^	1,093	4.0% (2.2–5.9)	750	29.7% (24.0–35.3)	25.6* (19.7–31.6)

*Statistically significant weighted change, p<0.001.

^†^ Legs or feet ulcers, amputation, lower vision, retinal damage, dialysis, heart attack, diabetic coma, feet pain.

^‡^ Cardiovascular Disease Risk Factors = smoking, obesity, dyslipidemia or arterial hypertension.

^§^ Depression, chronic obstructive pulmonary disease, asthma, muscle or bone diseases, neurological condition or cancer.

| (Σ compliance of evaluated indicators/ Σ occasions in which the indicator was evaluated) X 100.

^¶^ Defined by individualized hemoglobin HbA1c targets based on age and presence of complications (existing retinopathy, nephropathy, or cardiovascular disease). Measured in subsample (2006: n = 1,093; 2012: n = 750).

### Changes in the quality of the diabetes care process and glycemic control

Most of the process of care indicators showed improvement, although uneven and not always significant, with several indicators worsening (Table 2). Salient indicators with significant improvements were: detection of dyslipidemia (51.9 absolute estimated change, 71.4% relative improvement between 2006 and 2012); surveillance and detection of hypertension (17.4 absolute and 35.2% relative improvement between 2006 and 2012); and compliance with the number of recommended physician visits (16.0% relative improvement from 2006 to 2012). In general, the lowest percentages of compliance were related to the HbA1c test, prevention of complications (with even significant decrease in compliance in some cases like for eye exam), and non-pharmacological therapy (eating and exercise plan), which had a low compliance in 2006 (3.7%) and in 2012 (6.8%) (3.1 absolute estimated change, and 3.2% relative improvement). In contrast, the indicator with the highest level of compliance was drug therapy (85.5% in 2012). None of the adults with diagnosed diabetes self-reported compliance with all indicators.

**Table 2:** Level of compliance of process of quality of care indicators for adult with diagnosed diabetes: changes between 2006 and 2012.

	Indicators		ENSANUT 2006 (a)		ENSANUT 2012 (b)		Estimated change 2012–2006 (c)	Relative improvement 2012–2006
QUALITY VARIABLE	Information (questions in questionnaire)	Quality care criteria	N 2,965	% (CI 95%)	N 4,483	% (CI 95%)	(b)-(a)	[c/(100-a)] x100
**Monitoring and control of type 2 diabetes**								
Disease surveillance	How many times have you visited the doctor for the purpose of controlling your diabetes (without visits to the emergency room)?	“4” times or more per year	1,699	58.8 (58.5–59.2)	3,113	65.4 (64.9–66.0)	6.6* (5.9–7.3)	16.0%
Glucose monitoring	What tests did your doctor perform or order to check your glucose and how many times you took the test?	“HbA1c testing at least “2” times a year	110	3.7 (3.6–3.8)	316	7.7 (7.3–8.2)	4.0* (3.5–4.5)	4.2%
**Surveillance and detection of cardiovascular disease risk factors**								
Surveillance or Detection of arterial hypertension	Previously diagnosed hypertension: how many times have you checked your blood pressure? / Non diagnosed hypertension: Have you visited a preventive medicine service for detection of hypertension?	“1” time or more per month, in the last 12 months Yes	1,539	50.5 (50.2–50.8)	3,095	67.9 (67.4–68.3)	17.4* (16.8–17.9)	35.2%
Detection of Dyslipidemia	Have you ever had one blood cholesterol or blood triglycerides measured?	“yes” or “yes”	803	27.3 (27.0–27.6)	3,461	79.2 (78.7–79.7)	51.9* (51.3–52.4)	71.4%
Overweight / obesity detection	Have you visited a preventive medicine service to have an overweight/obesity detection?	“yes”	-	ND	1,455	33.2 (32.6–33.8)	-	-
**Prevention of vascular complications**		**Annual**						
Urinary protein Detection	What tests did your doctor perform or order to detect the presence of protein in your urine?	Urine microalbumin test	167	6.6 (6.5–6.7)	342	12.6 (11.9–13.3)	6.0* (5.3–6.7)	6.4%
Retinopathy detection	What preventive actions have you taken to avoid complications?	Checking of eyes	330	12.3 (12.1–12.4)	386	8.6 (8.1–9.0)	-3.7* (-4.1 to -3.3)	-
Diabetic foot detection	What preventive actions have you taken to avoid complications?	Checking of feet	241	9.4 (9.2–9.5)	593	14.7 (14.1–15.2)	5.3* (4.7–5.9)	5.8%
**Treatment**								
Non pharmaceutical	Do you actually take other treatments to control your blood sugar?	Eating and exercise plan	119	3.7 (3.6–3.7)	297	6.8 (6.5–7.4)	3.1* (2.8–3.4)	3.2%
Pharmaceutical	Do you actually take pills or insulin to control your blood glucose?	Pills, Insulin, Pills and insulin	2,467	83.3 (83.2–83.5)	3,922	85.6 (85.0–86.1)	2.3* (1.6–2.8)	13.8%

* Change 2012–2006 statistically significant,

p < 0.001.

Among patients who reported hypertension or dyslipidemia in addition to diabetes (Table 3), the increased use of services was noteworthy: 93.8% of respondents had their blood pressure taken at least once a month in 2012, a statistically significant relative improvement of 76.4% from 2006. Also noteworthy was the increase in the percentage of patients with drug treatment for dyslipidemia (79.5% in 2012, up from 76.9% in 2006, with a relative improvement of 11.3%). However, compliance levels were very low, with a significant decrease in compliance between 2006 and 2012, for non-pharmacological management (reduction of salt intake in those with hypertension: 4.1 percentage points decrease between 2006 and 2012; recommendations for fat intake in patients with dyslipidemia measured 14.6% in 2012, 13.3 percentage points less than in 2006).

**Table 3:** Quality of process of care indicators for adult with diagnosed diabetes and any cardiovascular disease risk factors and their level of compliance (2006, 2012).

	INDICATORS		ENSANUT 2006 (a)		ENSANUT 2012 (b)		Estimated change 2012–2006 (c)	Relative improvement 2012–2006
QUALITY VARIABLE (Medical care)	Information (questions in questionnaire)	Quality criteria	% response to the quality criteria				(b)-(a)	[c/(100-a)] x100
				% (95% CI)		% (95% CI)		
**Hypertension**			n 1,186		n 2,090			
Control	How many times do you take your own blood pressure or someone takes it for you	“1” time monthly or more	889	73.7 (73.5–74.0)	1,985	93.8 (93.6–94.2)	20.1* (19.8–20.5)	76.4%
Non pharmaceutical treatment	Do you actually take another treatment to control your blood pressure?	Lowering the consumption of salt?	116	11.8 (11.7–11.9)	183	7.7 (7.4–8.1)	-4.1* (-4.4 to -3.7)	-
**Dyslipidemia**			n 803		n 1,423			
Non pharmaceutical treatment	During the last 12 months have you received treatment for high blood cholesterol?	Diet for reducing the intake of fat or cholesterol?	204	27.9 (27.5–28.4)	194	14.6 (14.1–15.1)	-13.3* (-14.0 to -12.7)	-
Pharmaceutical treatment	Did you receive any treatment for high cholesterol? Older than 40 years with a given cardiovascular disease risk factor (tobacco use, arterial hypertension, overweight/obesity) or Has a doctor told you that you have or have had a heart attack, angina pectoris, cardiac insufficiency or another heart disease?	Medication or Pravastatine, etc. (statins)	625	76.9 (76.2–77.8)	1,142	79.5 (79.0–80.2)	2.6* (1.6–3.6)	11.3%

* Changes 2012–2006 statistically significant,

p < 0.001.

Overall quality estimated by composite indicators remained below 50% in 2012, yet showed a significant increase in relation to the 2006 estimates (Table 4). Overall quality for patients with diagnosed diabetes and cardiovascular disease risk factor was consistently higher than for those reporting only diabetes in both surveys (44.8% vs. 32.3% in 2006, and 48.3 vs. 40.3% in 2012).

**Table 4:** Quality of diabetes care: Percentages of compliance* and estimated changes between 2006 and 2012.

Composite Indicators	ENSANUT 2006 n = 2,965	ENSANUT 2012 n = 4,483	Estimated Change	Relative improvement 2012–2006
	Weighted % (95% CI) a	Weighted % (95% CI) b	(%) (95% CI) c	[c/(100-a)] x100
**Overall 1 (general)** 10 indicators for all adults with diagnosed type 2 diabetes^‡^	32.3 (32.1–32.4)	40.3 (40.0–40.6)	8.0^†^ (7.7–8.3)	11.8%
**Overall 2 (additional)** 4 indicators for adults with diagnosed type 2 diabetes and any cardiovascular risk factors	44.8 (44.6–45.1)	48.3 (48.1–48.4)	3.4^†^ (3.1–3.7)	6.2%
**Global** including all 14 indicators	33.1% (32.9–33.1)	40.8% (40.6–41.0)	7.7^†^ (7.4–8.0)	11.7%
**Estimated change 2006–2012—controlling for other factors** ^§^				
**% Global quality of diabetes care**	**Estimated change (%)**	**95% Confidence Interval**		**P value**
**Survey year** 2006 (1) 2012 (2)	5.8	5.6	6.1	0.000

* Compliance: (S compliance of indicators/ S occasions in which each indicator was evaluated) X 100.

^†^ Statistically significant, p < 0.001.

^‡^ Only 9 indicators were evaluated in 2006 because non- information for overweight/obesity detection.

^§^ Multiple linear regression: age, sex, time since diagnosis of diabetes, presence of complications due to diabetes, presence of cardiovascular risk factors, type of insurance and socioeconomic status.

The global quality of care indicator including all 14 indicators, changed 7.7 percentage points (from 33.1% in 2006 to 40.8% in 2012). This change remained significant after adjusting for age, gender, time of diagnosis, presence of complications due to diabetes, presence of cardiovascular disease risk factors, type of health subsystem, and SES (adjusted change: 5.8, 95%CI 5.6–6.1, p<0.0001).

### Factors associated with glycemic control

Both regression models, logistic and linear, revealed a significant association between the global quality of care composite indicator and glycemic control (Table 5).

**Table 5:** Association of quality of process of diabetes care and glycemic control.*

	Multiple linear regression		Logistic regression		
	Dependent variable: % HbA1c		Dependent variable: Glycemic control^†^		
	Beta coefficient	95% CI Beta coefficient	Odds Ratio	95% CI Odds ratio	P value
**Global quality of care** ^‡^	-1.29	-1.63 to -0.95	2.53	1.63–3.94	0.000
**Gender**					
Male	0.0		1.0		
Female	0.16	0.10–0.23	0.73	0.66–0.80	0.000
**Age**					
20–44	0.0		1.0		
45–64	-0.78	-0.87 to -0.70	3.11	2.82–3.43	0.000
65+	-2.10	-2.20 to -2.00	7.39	6.70–8.16	0.000
**Type of health subsystem**					
Social Security	0.0		1.0		
Seguro Popular	-0.17	-0.33 to -0.01	1.50	1.23–1.83	0.000
Other health services (private/others)	0.49	0.40–0.58	1.88	1.56–2.27	0.000
**Socioeconomic status (Quintile)**					
Q I	0.0		1.0		
Q II	-0.28	-0.42 to -0.14	1.15	0.97–1.36	0.09
Q III	-0.15	-0.25 to -0.04	1.06	0.88–1.28	0.52
Q IV	-0.19	-0.27 to -0.10	1.19	1.04–1.36	0.008
Q V	-0.23	-0.37 to -0.09	1.25	1.03–1.51	0.02
**Survey year**					
2006	0.0		1.0		
2012	-1.68	-1.75 to -1.61	9.96	8.19–12.1	0.000

* Changes controlling for others factors (time of diagnosis of diabetes, presence of complications, presence of CV risk factors).

^†^ Defined by individualized hemoglobin HbA1c targets based on age and presence of complications (existing retinopathy, nephropathy, or cardiovascular disease). Measured in subsample (2006: n = 1,093; 2012: n = 750).

^‡^ Global quality of diabetes care: compliance of indicators which are applicable (% of composite indicator compliance).

Adjusted OR in the logistic model for global quality in relation to glycemic control is 2.53 (95%CI: 1.63–3.94). The same significant association but with higher OR holds for the older age categories compared to the youngest category, higher SES compared to lower, and private and *Seguro Popular* affiliates as compared to other social security schemes. However an inverse relationship occurs when comparing females to males. Glycemic control is also significantly better in 2012 as compared to 2006 (OR: 9.96, 95%CI: 8.19–12.1). The model is adjusted by time since diagnosis, presence of complications due to diabetes, and presence of CV risk factors.

The linear regression model, with glycemic control expressed as % of HbA1c and global quality as a percentage of composite indicator compliance, shows the same consistent results: a significant association between global quality and level of HbA1c (β:-1.29, 95%CI -1.63 to -0.95), indicating that 1 percentage point increase in global quality of care is associated to a decrease of 0.95 to 1.6 percentage points in HbA1C, adjusted by all the other considered variables.

Logistic regression, included in the model for all simple quality care indicators as independent variables (data not shown in table), shows a significant association between the use of health services (expressed as ≥4 visits per year, OR: 1.51, 95% CI: 1.25–1.84) and glycemic control, and also better in those tested for HbA1c at least twice a year (OR: 2.0, 95% CI: 1.70–2.45). In addition, when compared to those receiving no drug treatment, the frequency of insulin use was significantly higher in uncontrolled patients treated either with insulin only (OR: 0.2, 95% CI: 0.16–0.39) or with insulin combined with oral hypoglycemic agents (OR: 0.07, 95% CI 0.04–0.12). Other factors associated to glycemic control were detection of hypertension (OR: 0.86 95% CI: 0.74–0.99), detection of dyslipidemia (OR 1.88, 95% CI: 1.61–2.18), and diet and exercise plan (OR: 0.31 95% CI: 0.19–0.52).

## Discussion

The results of this study show that despite the progress that Mexico has made in diabetes care, there is still considerable room for improvement. Global quality is still below 50%, and many of the relevant specific indicators have not improved significantly over the last six years. There is controversy regarding some indicators such as the appropriate number of physician visits and the benefit of insulin compared with oral hypoglycemic agents [33]. However, according to our findings, suboptimal performance affects most indicators, not only those that are potentially controversial. Our study highlights low compliance in the periodic consistency of HbA1c testing, non-pharmacological treatment, and other good practice indicators (e.g. ophthalmological examination or a eating and exercise plan) aimed at preventing complications. Pharmacological treatment for both diabetes and dyslipidemia achieved the highest compliance levels, which could contribute to better control and delayed onset of vascular complications; however, there still remains a significant percentage of patients without treatment (20%).

Among our study results, a significant increase in the prevalence of diabetes and its complications, poor compliance with prevention actions, and enhanced pharmacological treatment all suggest a reactive attitude by the health care system in general. This stresses the need to implement a comprehensive care model including all aspects of prevention, both primary and secondary, along with a more proactive attitude by healthcare providers.

Mexico has implemented specific programs that seek better and more holistic care for patients with diabetes [34–36], in addition to extending health provision coverage through the *Seguro Popular*. However, positive results from these efforts at the population level are still scarce, which may be partially related to the deficient quality of the actual care provided. In 2012, more than 85,000 people died in Mexico from diabetes-related causes [37], with an estimated avoidable decrease in life expectancy of eight years [5]. This problem is not unique to Mexico: in other countries, developed and developing, complications and costs of diabetes care continue to rise.

International studies show that good quality of diabetes care is difficult to achieve [38–42]. In this study, the quality of processes of care was the most important indicator. Individually, indicators of quality of care in diabetes in Mexico are similar in the international context. However, our data shows that deficiencies, mostly those related to preventive and therapeutic actions, in the case of Mexico are particularly relevant.

Insulin use is an important and controversial issue. It varies greatly among countries (more in developed than in developing countries) and with patient age (median use is 25%, but 50–70% in those younger than 45 years). In a comparison of 65 studies, the rate of insulin use among patients in Mexico was ranked among the lowest at <15% [43]. In this study, insulin use was associated to disease severity and poorer glycemic control. These seemingly reverse results are similar to those found in other studies [44]. It seems that insulin is most often used reactively when glycemic control has worsened and diabetic complications are already present. Detection of dyslipidemia was associated to glycemic control, but hypertension and non-pharmacological treatment (diet and exercise) were associated with poor glycemic control. Regarding the later finding, it seems that with the use of insulin a more complete treatment is provided among patients with uncontrolled diabetes. This finding reinforces the hypothesis of the need of a more proactive attitude focused on prevention.

Compliance with the number of physician visits and HbA1c testing were associated with glycemic control. Evidence regarding the regularity with which Type 2 diabetes patients need a medical review is still scarce, but at least one visit every three months is generally recommended [15,45]. Furthermore, there is consensus that HbA1c needs to be tested to monitor whether patients are being adequately treated or treatment adjustments are necessary [15–17, 29].

Glycemic control is the best indicator of the effectiveness of diabetes care, and is associated with fewer cardiovascular and renal complications [46, 47]. Some countries report up to 50% of Type 2 diabetes patients with glycemic control [48, 49]. The figure of only 30.5% in the 2012 survey in Mexico indicates an important gap in the health system’s performance: 4.8 million people (75% of adults with diabetes) are at increased risk of micro- and macro-vascular complications. The absolute improvement that we found (3.9% in 2006 to 30.5% in 2012) could have prevented complications such as stroke and amputations. The fact that this improvement is accompanied—and significantly associated with—an improvement in overall quality suggests that quality care is a major factor in the control of diabetes and related complications. In addition, given that older adults are more likely to achieve glycemic control, as observed in other studies [22, 44], it seems important to pay even greater attention to young adults.

Aggressive treatment targets for glycemia (such as HbA1c level <6.0% and <6.5%) in large randomized, controlled trials were associated with harmful outcomes [17], increased risk of severe hypoglycemia [50], and higher risk of mortality particularly in younger patients [51]. According to others authors [18], we suggested individualized glycemic targets based on recent consensus statements. These statements propose lower glycemic control targets for younger, healthier patients and more lenient thresholds for older patients with complications (defined as existing retinopathy, nephropathy, or cardiovascular disease).

Some limitations of this study should be mentioned, such the biases inherent in data obtained through surveys, residual confounding through unmeasured variables, the observational and transverse nature of its design, and possible differences among survey formats. However, the validity of the information obtained from patients in relation to other data sources such as medical records has previously been demonstrated in numerous studies [52, 53]. With regard to Mexico, compliance with quality of care indicators in our study is similar to the findings of another study that involved reviewing records [11]. This suggests that the information we analyzed is reliable. The cross-sectional survey design precludes any inference of causality, but comparisons between surveys are valid since the sample design and method of data collection are similar. It is noteworthy that the analysis of data using separate data files yielded similar results that the analysis using a combined data file, which is perhaps a preferred approach to assess changes controlling for others factors and to get more stable estimates [32].

Our study offers new insight into quality improvement strategies stratified by groups of patients with poor glycemic control and who may benefit from targeted interventions, in particular, younger adults. Tricco et al. [54] confirmed that multiple quality improvement strategies show improvement in diabetes care. Improving diabetes management should be included in interventions targeting the health system along with interventions targeting patients. Interventions targeting health care providers of chronic disease management seems to be beneficial only if baseline HbA1c control is poor.

However, few intervention studies have focused on addressing challenges faced by younger adult patients with Type 2 diabetes. Further research is needed to determine what interventions may be appropriate and effective for these younger patients, to accompany existing evidence that suggests that recommendations to diminish the high consumption of fat and sugar among young adults [55] may be beneficial, even if it is a challenge to change patient behavior and encourage healthy lifestyles.

In conclusion, diabetes remains a public health emergency in Mexico that continues to grow. Comprehensive policies and strategies to further improve the quality of diabetes care are needed, with more emphasis on preventive approaches and the non-pharmacological management of patients, and with increased proactivity among health professionals.
